# Pachymeningitis in Granulomatosis with Polyangiitis: A Case Report and a Review of the Literature

**DOI:** 10.1155/2013/840984

**Published:** 2013-08-06

**Authors:** Grigorios T. Sakellariou, Nicoleta Kefala

**Affiliations:** ^1^Department of Rheumatology, 424 General Military Hospital, 564 03 Thessaloniki, Greece; ^2^Department of Internal Medicine, 424 General Military Hospital, 564 03 Thessaloniki, Greece

## Abstract

Central nervous involvement, mainly with symptoms of cranial neuropathies, occurs in 2–8% of patients with granulomatosis with polyangiitis (GPA). Meningeal involvement, with persistent and severe headache as main manifestation and abnormal thickening and enhancement of the dural mater on postcontrast magnetic resonance imaging, is extremely rare. We present a case of pachymeningitis due to limited GPA, providing simultaneously a literature review.

## 1. Introduction

Granulomatosis with polyangiitis (GPA, formerly known as Wegener) is a rare, systemic disease of unknown etiology, characterized by necrotizing granulomatous inflammation and vasculitis, which in its classic form chiefly affects the upper and lower respiratory tracts and kidney [1]. Involvement of the nervous system is seen in about 23–54% of cases. The most common neurological manifestations are mononeuritis multiplex or, less frequently, distal symmetric sensorimotor polyneuropathies [2, 3]. Involvement of the central nervous system occurs in 2–8% of the patients with GPA and is commonly associated with dysfunction of one or several cranial nerves, most commonly the second, third, sixth, and seventh nerves. Meningeal inflammation, also known as hypertrophic pachymeningitis due to the typical presentation of thickening and contrast enhancement seen on magnetic resonance imaging (MRI), is extremely rare. We describe a patient with limited GPA with nasal lesions, otitis media, mastoiditis, sensorineural hearing loss, pachymeningitis, and positive cytoplasmic and proteinase-3 (PR-3) anti neutrophil cytoplasmic antibodies (ANCA). A literature review of pachymeningitis due to GPA is also provided.

## 2. Case Presentation

In January 2012, a 50-year-old man presented with nasal obstruction, a “clogged” sensation in his left ear, tinnitus, vertigo, and severe headache on the left parietofrontal area of the face.

The patient reported that the nasal congestion with fluid-thickened secretion started one year ago. After 3 months, he presented with a “clogged” sensation in his left ear with headache, which led to a diagnosis of serous otitis media. Myringotomy was performed, without resolution of symptoms. In August 2011, arthralgias, especially of the large joints (knees, elbows), was initiated, which resolved with low-dose prednisone for one month. In October 2011, the “clogged” sensation in his left ear and headache were deteriorated, and brain computed tomography (CT) was performed, which showed left mastoiditis. The patient was treated with antibiotics and nonsteroidal anti-inflammatory drugs, and after one month mastoidectomy was performed, without relief of symptoms. Ten days ago, he reported tinnitus, vertigo, further deterioration of headache, joint pain of his hands and foot, morning stiffness, fatigue, and low-level fever.

Physical examination revealed synovitis of bilateral wrists. There were no findings for cutaneous, ocular, neurological and pulmonary involvement. Otolaryngologic examination found perforation of the nasal septum with crusting and left otitis media. The audiogram revealed sensorineural hearing loss of the left ear. A nasal biopsy was performed.

Laboratory tests showed increased inflammatory markers [erythrocyte sedimentation rate (ESR): 72 mm, C-reactive protein (CRP): 28.9 mg/L], leukocytosis (WBC: 16.500 K/*μ*L), thrombocytosis (PLT: 533.000 K/*μ*L), positive rheumatoid factor (RF) [RF: 39.4 IU/mL (>20 IU/mL, positive)], IgG: 3.980 mg/dL, positive cytoplasmic ANCA (c-ANCA), at a titer 1/320, and PR-3 ANCA antibodies [PR-3 ANCA: 24.08 U/mL (>8 U/mL, positive)], and normal renal function tests (serum creatinine: 1.0 mg/dL, serum urea nitrogen: 35 mg/dL). Urine test was normal, and perinuclear ANCA (p-ANCA) and MPO ANCA antibodies were negative.

**Figure 1:**
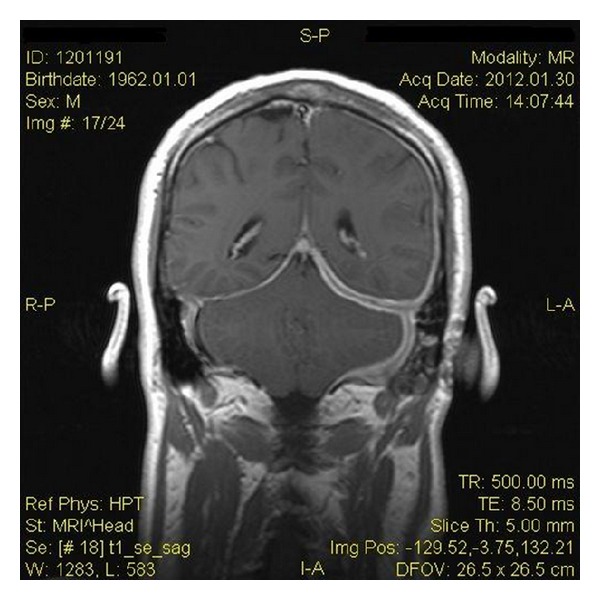
Gadolinium-enhanced coronal T1-weighted MRI scan showing pachymeningeal enhancement over the left temporal cerebral convexity, the left cerebellar convexity, and the left side of tentorium cerebelli. Also, there is left mastoiditis.

Brain MRI on postgadolinium T1 sequence revealed thickness and pathological enhancement of dural mater along the left hemisphere of brain and cerebellum, with expansion to tentorium (Figure 1). Also, the mastoid air cells on the patient's left side were opacified. Chest X-ray was normal.

**Figure 2:**
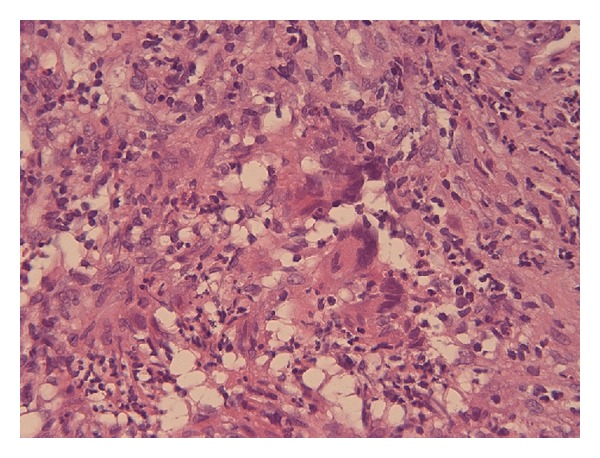
Nasal biopsy showing numerous multinucleated giant cells (haematoxylin-eosin staining).

**Figure 3:**
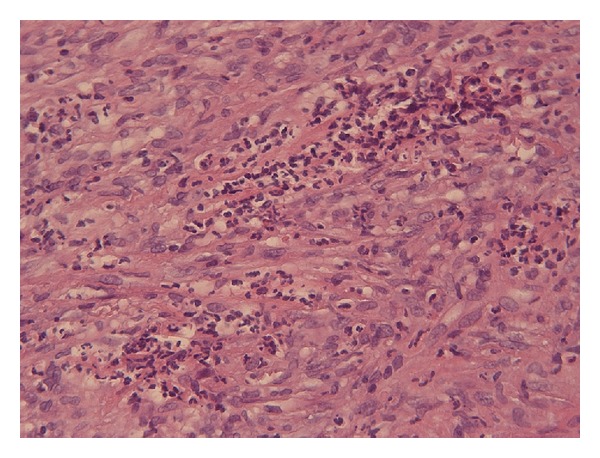
Nasal biopsy showing aggregate mainly consisting of neutrophils within and around blood vessel walls (haematoxylin-eosin staining).

Histological examination of nasal mucosa showed inflammation with presence of confluent polykaryocyte giant cells type Langhans (Figure 2) and vessels with lesions of type leukocytoclastic vasculitis (Figure 3), findings suggesting GPA.

A diagnosis of limited GPA with involvement of the upper respiratory tract (nose, ear, and sinus) and central nervous system (meninges and auditory nerve) was made. The patient initiated prednisolone 32 mg daily with tapering and monthly pulses, totally seven, of cyclophosphamide (CYC) 1.5 gr in combination with hydrocortisone 1 gr. There was prompt resolution of all symptoms, with remaining mild sensorineural hearing loss of the left ear, improvement of abnormal laboratory tests (reduction of inflammatory markers to normal level and resolution of thrombocytosis in the 1st month, and negativity of positive c-ANCA and PR-3 ANCA antibodies in the 6th month), and moderate resolution of pachymeningitis on repeated brain MRI. At present, the patient takes methotrexate (MTX) 20 mg weekly and prednisolone 8 mg daily. Because any effort for reduction of prednisolone at a dose lower than 10 mg/day has led to headache regression, we consider to treat the patient with rituximab.

## 3. Discussion

Meningeal inflammation is a rare manifestation of GPA. In two large series, meningitis was observed in none of 158 (0%) and 2 of 324 (0.6%) patients with GPA, respectively [2, 3]. Review of the English literature including case reports uncovered 54 patients with GPA and meningitis. Meningeal involvement more frequently occurs early in the course (within 6 months of onset) of clinically active, limited GPA [4, 5], as in our patient. However, in only 2 patients with GPA and meningitis, mastoiditis has been reported [4].

Persistent and severe headache is the predominant and almost always the first symptom of meningeal involvement of GPA [5]. Because headache is a common symptom in patients with GPA, due to chronic sinusitis or orbital disease, meningeal involvement may remain unrecognized for a long time. In most of the patients with meningeal disease neurological manifestations of cranial nerves have been reported, most commonly the second, third, sixth, and seventh nerves. Dysfunction of the eighth nerve rarely occurs [6, 7]. Seizures and encephalopathy have been described. In the majority of cases there were elevated inflammatory markers such as ESR and CRP. A positive serum ANCA, either cytoplasmic or perinuclear, is found in about two-thirds of patients. In accordance with these mentioned above, our patient had persistent headache as main symptom, dysfunction of cranial nerve, elevated inflammatory markers, and positive c-ANCA and PR-3 ANCA. Considering that there was improvement of hearing loss concomitant with imaging improvement of the meningeal inflammation, we suspect most likely a retrocochlear lesion such as fibrous entrapment of auditory nerve due to thickened and inflammatory dural mater than a cochlear lesion due to autoimmune process to be involved in the eighth cranial neuropathy.

The sensitivity of CT to detect meningeal disease is low, and in most cases the examination of cerebrospinal fluid (CSF) shows nonspecific abnormalities such as mild pleocytosis, consisting mainly of lymphocytes [8]. However, the widespread application of MRI has greatly facilitated early recognition and followup of patients with pachymeningitis. The typical finding on MRI is thickening and contrast enhancement of dural mater. Leptomeninges involvement is found less commonly [4, 5]. Two distinct MRI patterns have been described: (a) diffusely abnormal meninges unrelated to sinus or orbital disease; (b) focal enhancing thickening adjacent to sinus or orbital disease [9]. Meningeal biopsy has been reported in 26 cases and was performed because of either absence of involvement of other organs or tissues or nonspecific findings on extracranial biopsy [4]. The most common finding was necrotizing granulomatous inflammation, with limited or no signs of vasculitis. It is thought that the pathogenic mechanism of pachymeningitis in GPA is a remote granulomatous inflammation affecting only the meninges (diffuse dural thickening and enhancement on MRI), or, most frequently, the spreading of granulomatous tissue from the nasal or paranasal cavities and contiguously invasion to the adjacent structures including meninges (focal dural thickening and enhancement on MRI) [4, 10], as in our patient.

Early treatment of pachymeningitis in GPA is associated with improved outcome in terms of neurological recovery. A gratifying response to immunosuppressive therapy with resolution of headache, improvement or stabilization of cranial neuropathies and other neurological symptoms, reduction of ESR and CRP, and sometimes reversal of MRI abnormalities was observed in the majority of those with GPA-related pachymeningitis [5]. However, repeat brain MRI may show no [11, 12] or minimal [8] radiological improvement despite clinical recovery. This finding could represent residual postinflammatory fibrosis of dural mater [11, 12]. Therefore, to minimize treatment-related toxicity, the individual symptomatic response should be used to guide the rate of tapering the immunosuppressive drugs. Most cases, including our patient, initially received standard therapy with corticosteroids and CYC, either per os or intravenously on monthly courses, in doses similar to those used in generalized GPA, switching, after induction of remission, to oral MTX or azathioprine (AZA) for remission maintenance [5]. However, rituximab [12–14] or infliximab [15] has been successfully used in refractory cases.

In conclusion, pachymeningitis is undoubtedly a rare manifestation of active limited GPA. Heightened awareness, early diagnosis, and timely therapy are important to prevent permanent neurological dysfunction. If the clinical findings, ANCA results, MRI abnormalities, and extracranial biopsies are inconclusive or nondiagnostic of GPA, a dural biopsy may be necessary to confirm the diagnosis. Although there are no controlled studies, most patients with GPA-associated pachymeningitis respond favorably to treatment with corticosteroids and cytotoxic drugs (CYC, MTX, or AZA), particularly when such therapy is initiated early, before irreversible neurological damage.
